# The effect of proximity to grocery stores and the pandemic on parents’ and youths’ perceptions of eating habits in predominately African American rural communities

**DOI:** 10.3389/fnut.2024.1413208

**Published:** 2024-08-02

**Authors:** Amanda Williams, Sharlene D. Newman

**Affiliations:** Alabama Life Research Institute, The University of Alabama, Tuscaloosa, AL, United States

**Keywords:** eating habits, grocery stores, rural, COVID-19, African American

## Abstract

**Background:**

Rural communities have a disproportionately high level of childhood obesity along with high rates of food insecurity.

**Objectives:**

To assess the eating habits of youth in predominantly African American rural communities and assess the association between proximity to a grocery store and eating habits as well as how the COVID-19 pandemic may have impacted eating habits.

**Methods:**

203 youth and parents completed a 16-item survey. Analyses were performed to assess the effect of having a neighborhood grocery store as well as to compare responses between youth and parents.

**Results:**

Having a neighborhood grocery store was associated with increased consumption of vegetables (*F*(1,122) = 41.56) and fruits (*F*(1,121) = 65.05), decreased consumption of chips (*F*(1,125) = 79.51) and a smaller percentage of overweight youth (χ^2^(1,125) = 5.03). Parents underestimated the effect of the COVID-19 pandemic on youth eating habits (χ^2^ (1,198) = 17.88).

**Conclusion:**

Proximity to a grocery store significantly associated with eating habits and weight in the rural communities examined. Given the steady decrease in the number rural grocery stores over the past decade it is important to consider how to improve access to healthy foods in these communities.

## Introduction

The rate of obesity rate in Alabama is high (1) with high school students in 2019 in Alabama having an obesity rate of 17.2% (2). The guidelines for healthy consumption have varied over time but 21/2 servings of vegetables and 2 servings of fruit per day is currently recommended by the U.S. Department of Health and Human Services (3). The average American does not consume the recommended servings of either vegetables or fruit and the statistics are worse in rural communities. Rural areas, like most of Alabama, have been linked to poorer healthy food consumption (4, 5), and availability (6, 7). Given that eating fresh fruits and vegetables is associated with lower weight (8) and better health outcomes (9) it is critical that we have a better understanding of the factors that affect healthy food consumption.

Meeting the recommended daily servings of vegetables and fruits is difficult in unhealthy food environments. An unhealthy neighborhood food environment (NFE) is one with few supermarkets, limited availability of fruits and vegetables, high availability of high-calorie foods and sugar- sweetened beverages (6). Poor NFEs have been suggested to contribute to obesity and chronic disease as well as play an important role in establishing healthy eating habits for adolescents. For example, Li et al. (10) found that children are more likely to be overweight if their families frequently shop at convenience stores that do not have a broad selection of fresh vegetables and fruit, while children whose families have grocery stores that are more accessible are more likely to have a healthy weight. Additionally, Kepper et al. (11) found that grocery store availability within 4 miles increased fruit consumption. However, the literature is inconsistent regarding the effect of proximity to grocery stores and weight/health (12). For example, a recent meta-analysis revealed an insignificant association between proximity to a grocery store and weight (13).

Food insecurity, a household-level condition of limited or uncertain access to adequate food, is higher in rural (14.7%) and southern (14.5%) households compared to the U.S. average (12.8% in 2022) (14). A recent study reported that food insecurity in the Alabama Black Belt was 11 times higher than the national average in 2021 (15). Rural areas have unique access issues that contribute to food security (16–18). For example, transportation (e.g., a lack of public transportation and the cost of vehicle ownership) in rural areas make it difficult to access grocery stores or markets. There are also several low-income rural communities in the south with many households requiring a larger share of their total budget for food purchases compared to urban households (19). Also, the number of grocery stores in rural areas decreased steadily between 1990 and 2015 (20) while the number of dollar stores (with limited selection of fresh vegetables and fruit) increased (21). Low-income, minority communities have been severely impacted by this decrease in grocery stores. In a recent study examining a rural Alabama Black Belt county, the seven fast food stores and 20 out of 22 convenience stores were in majority African American communities; there were no grocery stores in the parts of the county where 85% of the population is African American (10). Conversely, the majority white community has a supermarket and two full-service restaurants. Therefore, some sectors of the population are more likely to have both food insecurity and live in unhealthy NFEs.

During the lockdowns of the COVID-19 pandemic youth nutrition changed. While several studies showed an increase in adolescent eating disorders during the pandemic (22), others showed improved eating habits with more homecooked meals, and increased consumption of fruits and vegetables and decreased consumption of fast food and junk foods (23). A disproportionate number of Black and Latino workers made up the essential workforce during the pandemic; therefore, while children were home due to school closures, many of their parents were still going to work decreasing the likelihood that these children had increased number of homecooked meals.

The goal of the current study was to assess the eating habits of youth in rural Alabama Black Belt communities. Childhood obesity in Alabama is high at 22.1% (24). We surveyed both the youth and parents in three communities. We hypothesized that youth were not consuming the recommended servings of vegetables and fruits and that the number of servings varied with the NFE (e.g., presence of grocery stores). We also hypothesize that obesity levels were associated with NFE and that youth eating habits worsened during the COVID-19 pandemic.

## Methods

### Participants

203 individuals participated in the study (see Table 1 for participant demographic information), including 76 children and 127 parents. Participants lived in rural towns with large African American populations. The study was approved by the University of Alabama Institutional Review Board.

**TABLE 1:** Participant demographics.

	Youth	Parents
*N*	75	128
Age	12.7 ± 1.9	
Gender	34(F)/40(M)/1(NA)	
**Race/ethnicity**		
African American/Black	69	122
Bi-racial	1	0
Hispanic	1	3
White	2	3
NA	2	0

NA, no answer, F, female; M, male.

### Procedures

The study is part of a larger community-based participatory research study designed to improve health literacy in rural communities. The team was composed of researchers, community navigators (CNs) and youth council directors (YCDs) in three rural predominately African American towns (see Supplementary materials for community characteristics). These CNs and YCDs were chosen because they were active in their communities and had knowledge of and access to a diverse range of residents (e.g., age, gender) and worked well with youth, respectively. The three community navigators completed human subjects training and were instructed on verbal consent as approved by the University of Alabama Institutional Review Board.

The team held community meetings and administered a community health survey (25) to learn about the health concerns of the residents. All communities identified poor eating and exercise habits as a potential cause of the poor health in their communities. Additionally, youth councils were developed in each community. To better understand the eating habits of the youth in the community a brief survey, described below, was distributed by the community navigators at community events (e.g., health fairs, health education events, town meetings, etc) to parents. The youth participants were recruited from the youth councils. Because of difficulties with internet access the surveys were administered using paper and pencil.

### Survey

**FIGURE 1:**
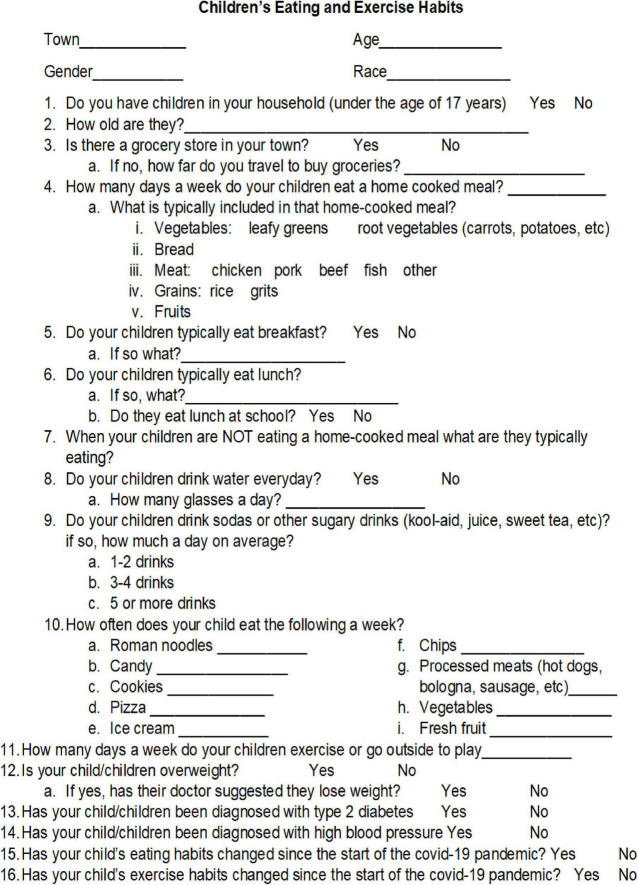
Parent survey.

The 16-question survey was designed to assess the typical weekly food consumption for youth in the rural communities of the Deep South (see Figure 1). In addition, information regarding access to a grocery store and how eating and exercise habits changed during to the COVID-19 pandemic. The survey was administered between September, 2022 through November 2022. The survey was anonymous; no identifying information was obtained. Additionally paper surveys were stored in a locked file cabinet in a locked file room allowing for confidentiality of the data.

### Analysis

Analyses were performed using SAS 9.4. Between subjects ANOVA and χ^2^ analyses were performed to examine response differences as a function of group (youth vs. parents) and proximity to grocery stores (see Supplementary materials for analysis by gender).

## Results

### Proximity to grocery stores

Table 2 presents the differences between responses of parents who have a grocery store in their community compared to those who do not. As shown, the majority of participants (∼85%) did not have a grocery store in their community. For those who do not have a grocery store in their community the average distance reported to a grocery store was 24.67 ± 7.7 miles. As shown, cooking and eating habits are poorer for children who do not have a grocery store in their community. Additionally, the rate of overweight youth is higher and the pandemic had a larger effect on eating and exercise habits for youth who did not have a grocery store in their community.

**TABLE 2:** Results by NFE.

	No grocery store	Grocery store	Statistics (*p*-value)
*N*	108	19	
Homecooked meals/week	3.62 ± 1.1	6.50 ± 0.89	*F*(1,114) = 92.45***
Sodas/day	2.97 ± 0.9	2.55 ± 1.03	*F*(1,125) = 6.38*
Roman noodles/week	2.92 ± 1.31	3.37 ± 1.77	*F*(1,125) = 3.2
Chips/week	3.16 ± 0.63	1.84 ± 0.69	*F*(1,125) = 79.51***
Vegetables/week	3.51 ± 1.12	5.75 ± 1.53	*F*(1,122) = 41.56***
Fresh fruit/week	3.33 ± 1.0	5.67 ± 1.68	*F*(1,121) = 65.05***
Days exercise/week	4.09 ± 1.41	5.60 ± 1.4	*F*(1,109) = 15.47***
Overweight	20%	0.00%	χ^2^(1,125) = 5.03*
Diabetes	6%	0.00%	χ^2^(1,125) = 1.19
Eating change due to pandemic	33%	6%	χ^2^(1,125) = 6.8**
Exercise change due to pandemic	31%	6%	χ^2^(1,125) = 6.15*

***p < 0.001; **p < 0.01, *p < 0.05.

### Parents versus youth

Table 3 presents the comparison between the youth and parents’ responses. As shown, there are differences in their responses to their unhealthy food consumption (e.g., sodas and chips) with parents underestimating the amount of chips and overestimating the consumption of soda. The most significant difference is that youth report a significantly higher rate of changes in both eating and exercise due to the pandemic compared to parents.

**TABLE 3:** Results by participant group.

	Child	Parent	Statistics (*p*-value)
*N*	75	128	
Homecooked meals/week	5.18 ± 2.08	4.05 ± 1.49	*F*(1,180) = 17.07***
Sodas/day	2.07 ± 1.04	2.90 ± 0.93	*F*(1,187) = 30.55***
Roman noodles/week	3.11 ± 2.16	2.99 ± 1.39	*F* < 1
Chips/week	4.14 ± 1.97	2.95 ± 0.79	*F*(1,197) = 36.23***
Vegetables/week	3.36 ± 1.83	3.81 ± 1.4	*F*(1,190) = 3.62
Fresh fruit/week	3.71 ± 2.1	3.62 ± 1.33	*F* < 1
Days exercise/week	5.26 ± 2.34	4.31 ± 1.49	*F*(1,175) = 10.83**
Overweight	17%	17%	χ^2^ < 1
Diabetes	0.00%	5%	χ^2^(1,199) = 3.56
Eating changes due to pandemic	60%	29%	χ^2^ (1,198) = 17.88***
Exercise changes due to pandemic	56%	28%	χ^2^(1,197) = 15.09***

***p < 0.001; **p < 0.01, *p < 0.05.

## Discussion

The major finding of the current study is living in close proximity to grocery stores was associated with increased consumption of vegetables and fruit, increased homecooked meals and decreased consumption of chips and soda. It also is related to lower reports of overweight youth. Additionally, the results showed that youth are getting far less than the recommended servings of vegetables and fruit per week and that parents reported a significantly smaller effect of the pandemic on their children’s eating and exercise habits than youth.

The data presented show that youth in predominantly minority rural communities, regardless of whether there is a grocery store, are failing to consume the recommended servings of vegetables and fruit. They are consuming far less than the recommended 21/2 servings (17.5 servings/week) of vegetables and 2 servings (14 servings/week) of fruit a day. The numbers are exacerbated in communities without a grocery store. There are several explanations for the finding. In a recent study of a similar population by Holston et al. (16) it was reported that the majority Black rural Louisiana residents have inadequate access to food in their community and experience barriers (e.g., price gouging, transportation issues) when acquiring food within and outside of the community. A second explanation is food insecurity such that low-income families simply cannot afford the cost of fresh vegetables and fruit.

The current study also found that the NFE was associated with the percentage of youth who were overweight. The research showing a relationship between the community neighborhood food environment, particularly neighborhood grocery stores (12, 13), and health have been inconsistent. Kraft and colleagues (6) in their review of the literature argue that it is important to examine the effect in different subpopulations. They found only one study examining the relationship between health and food environment in rural youth (26). The current study adds to the literature on the effect of neighborhood food environment by focusing on predominately Black, rural youth. The findings show families had more homecooked meals and youth ate more fruits and vegetables a week than youth without a community grocery store. The youth also consumed less soda and chips all of which likely contributed to a smaller percentage of the youth being overweight.

The youth reported that the pandemic affected both their eating and exercise habits. Several studies have reported that the COVID-19 pandemic, from the lockdowns and school closings to the high death rate particularly in rural Black communities in the South, negatively affected the well-being of youth (27). The current study found that the pandemic was associated both youth’s eating habits and physical activity. As mentioned earlier, a disproportionate number of Black and Latino workers made up the essential workforce and continued to leave the home to go to work while their children were out of school. As a result, these households did not see the positive improvements in diet reported in the literature (23). This may explain the differences in the youth and parent perspectives - a higher percentage of the youth reported changes in both eating and physical activity than parents.

Another important finding is that parents who do not live near a grocery store reported that their children had changes in eating and physical activity that they linked to the pandemic at a higher rate than those who live near grocery stores. What has replaced local grocery stores in many rural communities is the dollar store (21, 28). These dollar stores, like convenience stores, have highly processed, foods (chips, cookies, candy, etc) (28). Without access to fresh fruits and vegetables in grocery stores, it may be that youth snack more on junk food and that may have increased during the pandemic. Support for increased snacking on junk food is seen in the greater consumption of chips reported in the no grocery store group compared to the grocery store group. It is important to look more closely at these pandemic differences.

### Limitations

There are some limitations to the study that should be addressed. First, the sample size is relatively small, particularly the group that has a grocery store in the community. We also failed to obtain income and employment information or access to free or reduced school meals that may help explain results. Second, the survey data is all self-report reducing its reliability. Finally, more information regarding how eating and physical activity changed due to the pandemic would be helpful in better understanding results.

## Conclusion

The current study adds to the literature investigating the effect of NFE on the health of youth. We examined an understudied population, rural African American youth and found that not only were they not consuming the recommended servings of vegetables and fruit, but that the amount was associated to their proximity to a grocery store. Additionally, we report that NFE was associated with the percentage of overweight youth. The results beg for a larger, more comprehensive study to better characterize the relationships reported.

## Data availability statement

The raw data supporting the conclusions of this article will be made available by the authors, without undue reservation.

## Ethics statement

The studies involving humans were approved by the University of Alabama Institutional Review Board. The studies were conducted in accordance with the local legislation and institutional requirements. The ethics committee/institutional review board waived the requirement of written informed consent for participation from the participants or the participants’ legal guardians/next of kin because there was minimal risk associated with the project.

## Author contributions

AW: Conceptualization, Formal analysis, Writing – original draft. SN: Conceptualization, Data curation, Formal analysis, Funding acquisition, Supervision, Writing – original draft.
