# Supplementary material for: The effect of proximity to grocery stores and the pandemic on parents’ and youths’ perceptions of eating habits in predominately African American rural communities
Source: Front Nutr. 2024 Aug 2;11:1413208. doi: 10.3389/fnut.2024.1413208 (PMC11329938; doi:10.3389/fnut.2024.1413208)
Supplement: Supplementary file 1 [file Table_1.DOCX]

**Supplementary Materials**

**Community characteristics**

| **Town** | **Population** | **% African American** | **Per capita income** | **SV Index** |
| --- | --- | --- | --- | --- |
| Community 1 | 1,859 | 85.5% | $19,618 | 0.8283 |
| Community 2 | 2,239 | 88.4% | $14,872 | 0.9537 |
| Community 3 | 2,847 | 64.2% | $10,782 | 0.9175 |

Note: SV index= social vulnerability index.

***Gender differences***.

|  | **Male** | **Female** | **Statistics (p-value)** |
| --- | --- | --- | --- |
| **N** | **40** | **34** |  |
| Homecooked meals/week | 4.93±2.5 | 5.45±1.46 | F=1 |
| Sodas/day | 2.11±1.11 | 2.03±1.0 | F<1 |
| Roman Noodles/week | 2.84±2.34 | 3.38±1.91 | F=1 |
| Chips/week | 3.53±1.9 | 4.94±1.78 | F(1,68)=10.17** |
| Vegetables/week | 3.17±1.76 | 3.70±1.82 | F(1,61)=1.45 |
| Fresh fruit/week | 3.09±1.96 | 4.50±1.93 | F(1,65)=8.81** |
| Days exercise/week | 5.15±2.49 | 5.40±2.24 | F<1 |
| Overweight | 24% | 9% | χ^2^(1,69)=2.85 |
| Diabetes | 0.00% | 00% | n/a |
| Eating change due to pandemic | 58% | 64% | χ^2^ <1 |
| Exercise change due to pandemic | 46% | 70% | χ^2^(1,69)=4.02* |
